# Subjectivity in Entrustment Decision-Making in the Clinical Workplace: Can We Retain Its Legitimacy But Avoid Bias?

**DOI:** 10.1007/s40670-025-02539-w

**Published:** 2026-01-27

**Authors:** E. F. Wilma Kleijer, Inge A. Pool, Marieke J. Schuurmans, Olle ten Cate

**Affiliations:** 1 Utrecht University, Utrecht, The Netherlands; 2 Isala Academy, Zwolle, The Netherlands; 3 University Medical Center Utrecht, Utrecht, The Netherlands; 4 Education and Training, WIOO UMC Groningen, Groningen, The Netherlands; 5 Utrecht Center for Research and Development of Health Professions Education, University Medical Center Utrecht, Utrecht, The Netherlands; 6 Academy, University Medical Centre Utrecht, P.O. Box 85500, Room 4.66, 3508 GA Utrecht, The Netherlands

**Keywords:** Enstrustment decision-making, Clinical workplace, Legitimate subjectivity, Bias

## Abstract

After decades of emphasis on objectivity in assessment, the awareness of the significance of expert judgment in the clinical workplace has led scholars to acknowledge the value of subjectivity in such assessment. With the advent of entrustment-focused assessment, validity questions led us to examine subjectivity as a construct and to explore the role of bias within expert judgment. In this commentary, the authors propose a conceptual model of subjectivity that distinguishes implicit and explicit bias, as well as systemic and individual-focused bias, not to provide solutions to abandon bias, but to raise awareness of its conceptual role in entrustment decisions.

## Introduction

Expert judgment is inherent in the assessment of learners in the clinical workplace and brings differences between experts along [1, 2], which may be considered a form of subjectivity. While this has been discussed with regards to competence evaluation, in this commentary we highlight the nature of this subjectivity related to entrustment decisions, and argue that there is a legitimate and important component, which should be disentangled from unwanted bias. Our goal is to analyze and define what subjectivity in expert judgment for entrustment decisions in the clinical workplace means, and how bias may be distinguished from legitimate subjectivity. We aim not to provide ready solutions to eliminate bias, but to present a conceptual model of subjectivity in entrustment.

The clinical environment is the context in which much of the learning occurs for aspiring health professionals [3]. Learners acquire practical skills and link theoretical knowledge to practical application under the supervision of professionals [4, 5]. Effective assessment is critical to safeguard both professionals and patients against inadequate practice [6, 7].

The way the quality of assessment is being viewed and assured has changed [1]. In the 1960 s, multiple-choice tests were introduced in health professions education, as a response to common oral assessment practices, often perceived as subjective, unreliable, and biased [1, 8]. Assessment research and development focused on standardizing and objectifying assessment in health professions education. In this early era, psychometric scales categorized learners as competent, not competent, or in between [1]. The pursuit of reliability and objectivity characterized all assessments, transforming real-world phenomena into numbers. This applied to knowledge-based exams, as well as to so-called objective, structured examinations of clinical skills in simulation settings [2]. When, in the 1990 s, assessment attention shifted to authentic contexts, workplace-based assessment (WBA) tools were developed, and transparency and outcomes-oriented clinical performance assessment was stimulated with the introduction of competency-based health professions education [8]. Without returning to traditional unreliable clinical assessment practices, there was a reappraisal of the role of human judgment in the assessment process. The role of preceptors with expertise about the clinical context, assessment conditions and methods, and interpretation of observations became more important [1], as well as detailed performance criteria helping preceptors to evaluate learners’ performance [9]. This must be reference Norcine & Borch, see refence 9 in the reference list. The dominant pursuit in WBA remained optimizing reliability and validity, in analogy to written assessment [10]. However, educational and psychometric scholars started questioning that analogy, as test conditions cannot be standardized in the clinical environment, and standardization is necessary to determine reliability [1, 11]. While canonical knowledge and skills can be defined in generalized standards and assessed in standardized tests, contextual competence requires adaptability to an environment that is never the same [12]. Even with the most sophisticated WBA instruments, assessment in healthcare settings is always affected by experts’ subjective impressions, considerations, experiences, and values [13, 14].

### Entrustment Decision-Making

The introduction of the concept of entrustable professional activities (EPAs) in 2005 started a movement toward entrustment decision-making as an approach to assessment in the workplace [15, 16]. In entrustment decision-making, trust and risks are fundamental concepts. Trusting is a dynamic process between key players in health professions education, including patients and professionals but, similarly, between supervising preceptors and professionals in training [17]. Preceptors must frequently determine how much autonomy to grant learners in patient care, weighing potential risks for patients. They need to be able to identify whom they can entrust with an activity and whom not (yet) [18]. Preceptors’ intuitions or gut feelings about learners play a role in guiding decisions about their readiness to take care of patients unsupervised. Different preceptors may have different gut feelings that guide their judgment about learners in roughly similar situations [8, 10, 19].

Entrustment decision making distinguishes ad hoc and summative decisions. In competency-based programs that use entrustable professional activities (EPAs), summative entrustment decisions are formalized qualifications to act at a specified level of autonomy, resembling a license to practice, but restricted to one EPA. Following a programmatic assessment paradigm, summative entrustment decisions, typically made by a team, are based on multiple data points, including a series of ad hoc decisions and their evaluations. Summative decisions are high stakes and need to be as rigorous as possible; ad hoc decisions are usually lower stakes, but nevertheless essential for learner experience. As their evaluations, documented in a learner portfolio, eventually contribute to summative decisions, it is worth exploring how these are affected by preceptor subjectivity in a legitimate or a biased way.

Conventional competency-based assessments are designed to “objectively” report performance after an observation [20]. Entrustment-supervision scales require real-world prospective judgments of readiness for autonomy. Entrustment decisions (e.g., asking a learner to “take over,” or deciding that direct supervision “is no longer needed”) belong to daily practices in teaching hospitals. Such decisions require preceptors’ thoughts on learner trustworthiness, ability, and needs [21, 22]. Entrustment-based assessments expand the focus beyond evaluating learners’ competence to allow for learner progress decisions, to assessing their readiness for a level of clinical autonomy, incorporating trustworthiness and contextual judgment, ultimately affecting patient wellbeing and appropriate clinical care [23].

**Fig. 1:**
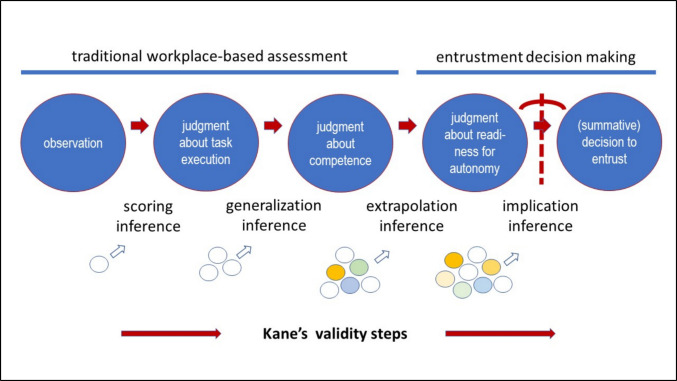
Kane’s validity framework of inferences applied to workplace-based assessment and entrustment decision making

The difference between the more traditional competency-based versus the entrustment-based assessments can be explained using Kane’s validity framework (Fig. 1, adapted from Touchie et al. [23]). Inferences, based on assessment information, are needed to arrive at conclusions about learners. Kane distinguishes four inferences—scoring, generalization, extrapolation, implication—the first two of which are most common in education [24].

The higher in Kane’s chain of inferences, the more holistic the decision becomes, and the more comprehensive information is needed. Both Kane and Messick stress that inferences require arguments that support the validity of the use of a score. If the use of assessment must support the decision to entrust trainees with patient care, consequential validity is at stake, which requires a higher-level inference than most summative test scores in education. The validity of such a rich prospective decision may require more information than can be gleaned from direct observations alone [25]. While ad hoc entrustment decisions may rely on immediate contextual judgment, summative entrustment decisions require multiple sources of performance data and input from multiple preceptors to support their validity [26]. The better quality of each data point, the more valid the summative decision. After each ad hoc decision, a preceptor may report with a recommendation for readiness for a specific supervision level. Such higher-order inferences involve deeper thought processes and likely more extensive subjectivity, personal estimations of future readiness based on preceptor experience, than direct performance observations.

### Subjectivity

Subjectivity is “the fact of being influenced by personal ideas, opinions or feelings, rather than facts” (Oxford Dictionary). In terms of validity, the “subjective” influence on entrustment decisions may contain a construct-relevant component (“legitimate subjectivity”) and a construct-irrelevant component (“bias” or, in terms of the Oxford Dictionary, “inclination or prejudice for or against one person or group, especially in a way considered to be unfair”). Much of the performance in health care workplaces cannot, however, be fully qualified in “objective facts” about learners that are either correct or not.

Educational scholars in the health professions are increasingly convinced that subjective expert judgments should have a place in assessments of learners. At the same time, however, unwanted bias is undesirable [8]. Bias, which triggered the development of the objective assessment movement from the 1960 s, can manifest in various areas in the clinical workplace, including the learning environment, assessment instruments, preceptors, and evaluators of assessment [21]. It would be very useful to disentangle bias, i.e., illegitimate subjectivity in assessment, from legitimate subjectivity.

**Fig. 2:**
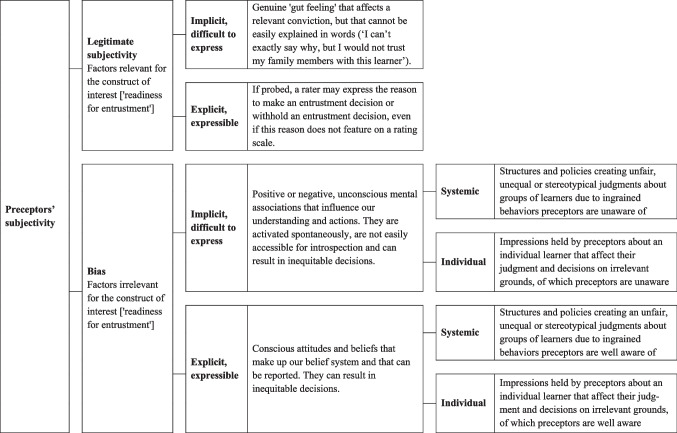
Conceptual framework to disentangle legitimate subjectivity and bias in entrustment decision-making

While acknowledging that the two do not reflect a clear-cut dichotomy, we developed a conceptual framework that attempts to disentangle legitimate subjectivity and bias in entrustment decision-making (see Fig. 2).

### Inter-Observer Variability

Before analyzing legitimate subjectivity and bias more in depth, we will address the significance of inter-observer variability.

Preceptors are indispensable “instruments” in workplace-based assessment (WBA) of trainees. However, when multiple preceptors give ratings, even of the same situation and same learner, inter-observer variability is common [27]. Generalizability studies using large data sets from WBA have consistently shown that a large proportion of variance in scores is explained by preceptors; often larger than by learners being assessed [28]. Training of preceptors can reduce this variance component somewhat, but cannot exclude it [29].

Inter-observer variability has long been regarded as undesirable, construct-irrelevant error [27, 30] to be corrected, including bias caused by a preceptor’s clinical and educational expertise, their idiosyncrasy, strictness or leniency, bias, and variable frames of reference [14, 31]. What if we start seeing this variability as relevant and legitimate [32, 33] and not as a problem to be corrected psychometrically [34]? Inter-observer variability in judgments hardly ever implies that there is one “correct” perception, even hypothetically. Different and sometimes contradictory interpretations might exist simultaneously, and all may serve as useful assessment information, [35] simply because preceptors are experts who bring their personal perception and expertise to judgment [14].

Expert judgment involves experienced professionals making informed decisions based on their interpretation of observed behaviors, contextual cues, information from various sources, and their own frame of reference [19, 35]. It complements standardized (or “canonical”) knowledge and skills assessments, providing a more comprehensive evaluation of a learner’s readiness for entrustment [12, 21]. Research has identified key factors that influence entrustment decisions, including capability, integrity, reliability, humility, and agency [25]. Subjective expert judgment refers to preceptors’ personal evaluation and interpretation of learners’ performance across these dimensions. These evaluations often rely on high-level semantic qualifiers (e.g., demonstration of expertise, personal or professional credibility), used by preceptors to articulate their narrative for judgment of readiness for learner entrustment with care tasks [19, 36]. Implicit subjective expert judgment may not always be easily expressible in words or moldable into predefined categories of a proficiency checklist. This type of judgment is nonetheless key to determining a learner’s readiness for entrustment with a critical task or a license to practice. Implicit judgment is often tacit in nature and can be more challenging to surface explicitly [37].

### Gut Feelings in Entrustment Decision-Making

Entrustment implies a willingness to be vulnerable to another party who cannot be fully monitored or controlled, which is a general feature of trust [38, 39]. While preceptors can often articulate reasons for their entrustment decisions when pressed, these decisions frequently involve rapid judgments based on complex, contextual factors that are difficult to fully capture in standardized documentation and may be qualified as subjective, yet legitimate. Preceptors may use assessment tools and observations to confirm their holistic impressions. There is an analogy with clinical decision making [40, 41]. Just as diagnostic reasoning involves rapid pattern recognition in experienced clinicians, [40, 41] preceptor expert judgment relies on knowledge about the learner, recognizing and interpreting contextual cues, and identifying critical variations in the assessment task, and on implicit comparisons with earlier experiences with learners [34]. As experience in supervision increases, preceptors will make entrustment decisions in a more non-analytical manner, being triggered by a gut feeling if learners deviate from the expected pattern [19]. Ad hoc entrustment decisions are rapid, and gut feelings about the situation may weigh in. If analyzed, such decisions are affected by a myriad of variables, including those within the learner, the supervisor, their relationship, the context, and the task to be entrusted [42]. Learner features may even include seeming paradoxical behaviors, such as “humility” (asking for help and feedback) and “agency” (proactive behavior) [25]. Preceptors and trainees must navigate such expectations, and supervisory experience and acquaintance with the trainee may affect the decision, as well as power dynamics in the clinical context, space for learner autonomy, and patient safety. That may not be easy initially.

However, unlike diagnostic reasoning where clinicians receive extensive formal training, preceptors often make entrustment decisions without specific training in this skill. This suggests the need for targeted training programs to help preceptors recognize factors influencing their judgments and develop more systematic approaches to entrustment decision-making. Many of these features are not easily captured in assessment forms, but add to the holistic picture of the learner and may be characterized as “subjective” impressions, which are important for entrustment decisions.

### Bias — Systemic Versus Individual, and Implicit Versus Explicit

Bias has different meanings. It is important to distinguish between bias as a measurement concept in psychometrics and bias as social discrimination, though both can negatively impact fair assessment. In psychometrics, it has been defined as a statistical concept that refers to the systematic over- or underestimation of true scores in a population, that is, as an undesirable property of a measurement instrument. Bias arises when scores for the construct of interest in one subpopulation differ from those in another subpopulation, or when the relationship between the independent and dependent variables is distorted by a third variable, such as sex, age, or race [43]. Systemic bias, interfering with the need for diversity, equity, and inclusion of learners, has received much attention in recent years. Less often discussed is bias stemming from rater opinion about an individual based on construct-irrelevant information (such as learner characteristics unrelated to clinical performance, like gender, race, or personal appearance, as well as potentially misleading anecdotal information) [44]. Tweed et al. found that raters on OSCE stations would alter their score for a trainee that was initially based on several credible sources, if a supplemental anecdote was provided [45]. Individual bias is not caused by systemic prejudice, but by construct-irrelevant influences such as individual interactions or overrated fragmentary information.

Preceptors should be aware of the negative effects of bias on healthcare education and assessment [21, 46] with bias defined as “a strong feeling in favor of or against one group of people, or one side in an argument, often not based on fair judgment” [47]. Systemic bias stems from innate human tendencies to categorize information to facilitate interpretations and inferences and occurs when assessments are negatively influenced by learner characteristics, such as gender, race and ethnicity, religion, sexual orientation, ability status, and socioeconomic status [48] and refers to prejudices and stereotypical beliefs which are activated spontaneously [49]. Bias may also stem from hearsay about individual learners, and the undue weight of prior performance of a learner [50]. Prejudices relate to the negative attitudes, feelings, or impressions a person forms about other individuals or groups, often in advance, and not based on actual experience with the individuals concerned. Stereotyping refers to purely cognitive (not motivational or emotional), rigid, and generalized beliefs about a specific group of people [21, 51]. Prejudices and stereotyping become visible in discriminatory, unintentional, or intentional behavior [49, 52].

Bias can also be distinguished into implicit and explicit categories. Implicit biases can be positive or negative, and are unconscious mental associations that influence our understanding and actions. They are activated spontaneously and are not directly accessible for introspection [21, 49, 53]. No one is immune to implicit biases despite frequently heard beliefs that decisions can be fully objective [52] Explicit bias refers to consciously held and easily expressed attitudes. They are part of our belief system and can be reported [52–54].

### A Model of Subjectivity in Workplace-Based Entrustment

This brings us to a model of subjectivity that can be analyzed in components (Fig. 2).

## Discussion

Subjectivity in assessment is often equated with bias. Our key message is to create a more nuanced view of subjectivity in the context of entrustment decisions in the clinical workplace.

Bias, in the psychometric sense, is a deficiency of the measurement instrument. In workplace-based assessment, however, the rater *is* the instrument and is more important than any form to document a judgment. Raters not only differ from each other, but may differ within themselves based on context, mood, thoughtfulness, recent experiences, et cetera. The question then is, can legitimate subjective judgment be disentangled from unwanted bias in rater judgment?

This question becomes even more prominent in ad hoc entrustment decisions (such as “can I leave the trainee alone?”[55]), which require simultaneous consideration of multiple competence dimensions, with their relative importance varying according to specific circumstances (Fig. 1). Still, ad hoc entrustment decisions are momentary and can be reversed (“tomorrow’s decision may be different”), and the risks of a wrong decision are less serious than those of summative entrustment decisions, leading to more permanent privileges and increased autonomy.

Our model is primarily a thought exercise and not meant to provide a solution to optimize judgment and entrustment decisions. Yet, we can speculate what measures may be taken to improve judgments in the clinical workplace and entrustment decision-making, while including legitimate subjectivity and avoiding bias.

Becoming aware of bias in subjectivity is the first step in addressing it [48, 56]. Rater training programs that cover the nature of implicit and explicit biases, their impact, and strategies to mitigate them, and that target preceptors’ skills (e.g., developmental feedback skills, interpersonal and motivational skills), as well as awareness of values and beliefs associated with performance appraisal [14, 34]. Alternative approaches may include mentorship programs for supervisors, contextual reflection, and knowledge reorganization strategies that help preceptors systematically apply relevant information during entrustment decisions [57, 58]. Explicit bias, characterized by conscious attitudes and beliefs, is easier to detect than implicit bias, which operates unconsciously and is more challenging to identify [20]. Tools like the Implicit Association Test (IAT) can help preceptors uncover their biases, making self-awareness the first step toward conscious change, [59] critical thinking and reflection on one’s assumptions and assumptions of others [34, 46].

Besides raising awareness of bias and subjectivity, improving assessment practices can help distinguish bias from legitimate subjectivity. Aligned with programmatic assessment [60], we suggest that summative decisions should be based on multiple and varied interactions and observations, and time to capture valid and sufficiently holistic impressions about learners [33]. Information to ground summative decisions of entrustment has been summarized as stemming from four sources: direct observation, longitudinal monitoring, structured conversations with the learner, and product evaluation (i.e., results of care provided) [61].

Next, for important decisions with the nature of certification, stakes are high and validity must be optimized. Here is where team decisions have been strongly recommended. Summative decisions should be made after team deliberations, in which legitimate subjective input outweighs input based on bias. Combining input from different information sources is not meant to reach an “objective” judgment or decision. Rather, it is because multiple interpretations of a learner’s performance can be equally valid, providing a comprehensive report of competencies and situation-specific behaviors, emphasizing the need for holistic, inter-subjective assessments and consensus decisions [14]. Rather than restricting assessor variability through checklists, reducing tasks into predefined observable subcomponents only, which limit assessors in making their own expert judgments, [34] narrative assessment information should also be embraced because of the variation they allow. Faculty and learners’ assessment roles should be explicitly defined [20]. We speculate that team deliberations to arrive at summative decisions will tend to decrease unwanted bias, but this is an area of investigation. By analyzing learners’ performance contextually and making informed judgments based on preceptors’ expertise and experience, the quality of assessments can be improved [34].

We do not pretend that our model cannot be developed further. There may be other factors that affect entrustment decisions in a construct-irrelevant way other than systemic and individual bias. Here is an example. A precepting supervisor, knowing a learner well and judging that this learner has the full capacity and the right attitude to perform a clinical task well and safely, may still withhold an entrustment decision, expecting that their own superior, e.g., a head of department, would not allow this. In terms of the validity of the decision, a construct-irrelevant factor (at least not related to the competence to act) is added to affect the decision. This could be hierarchical power dynamics, but could also be legal restrictions. Clearly, those factors may apply differently in a different context or country, even with, theoretically, the same learner and preceptor. In other words, this limitation to the model makes it a starting point to think about entrustment decision making.

## Conclusion

“Objective” workplace-based assessment, if defined as yielding fully reliable decisions, free from subjectivity and bias, is arguably a fiction. Expert judgment is not only unavoidable; it should be valued as important contributing to rich decisions. This subjectivity may include bias, which should be avoided, but subjectivity also includes an important, legitimate component of information that may get lost in attempts to restrict assessment to predefined scales and forms. To address the distinction between legitimate subjectivity and unwanted bias, our model may serve as a starting point for discussion.
